# Tyrosine Hydroxylase Deficiency: From Clinical and Therapeutic Features to Pathogenetic Considerations

**Published:** 2026-10-07

**Authors:** YOSHIAKI FURUKAWA

**Affiliations:** 1 Department of Neurology, Juntendo Tokyo Koto Geriatric Medical Center, Tokyo, Japan; 1 Department of Neurology, Juntendo Tokyo Koto Geriatric Medical Center, Tokyo, Japan; 2 Department of Neurology, Faculty of Medicine, Juntendo University, Tokyo, Japan; 2 Department of Neurology, Faculty of Medicine, Juntendo University, Tokyo, Japan

**Keywords:** tyrosine hydroxylase gene (*TH*), dopa-responsive dystonia (DYT5b), motor development delay, striatal dopamine deficiency, levodopa therapy

## Abstract

Tyrosine hydroxylase (TH) deficiency is a rare metabolic disorder caused by biallelic pathogenic variants in the TH gene (*TH*). More than 95% of *TH* variants can be detected by usual sequence analyses and gene-targeted deletion/duplication analyses are necessary to identify gross abnormalities (e.g., exon deletions). The enzyme TH, a tetrahydrobiopterin (BH_4_)-dependent monooxygenase, catalyzes the rate-limiting step in catecholamine biosynthesis. Thus, symptoms result from dopamine deficiency and subsequent noradrenaline/adrenaline deficits, and most patients demonstrate positive response to levodopa. The phenotypic spectrum of this treatable disorder is extremely broad. Although the underlying mechanisms of phenotypic heterogeneity are still unknown, recent human and experimental data have indicated that 1) there is no dopaminergic neuronal degeneration in TH deficiency, 2) mildly affected patients may develop dopa-responsive dystonia through dysfunctional D1 dopamine receptors due to congenital dopamine deficiency and insufficient postnatal dopamine increases in the striatum, and 3) severely affected patients may demonstrate remarkable motor development delay in infancy and hypersensitivity to levodopa therapy (leading to levodopa-induced dyskinesias) caused by profound brain dopamine deficit and consequent abnormal synaptic and brain maturation. It is known that, in the normal human brain, development of the nigrostriatal dopaminergic system is incomplete at birth and striatal dopamine, TH, and its cofactor BH_4_ concentrations significantly increase during postnatal development. Therefore, dopamine deficiency should be corrected by appropriate treatment with levodopa (e.g., very low initial doses for severely affected cases to avoid intolerable levodopa-induced dyskinesias) as soon as genetically diagnosed or upon the appearance of characteristic symptoms of TH deficiency.

## Introduction

Tyrosine hydroxylase (TH) deficiency is a recessively inherited metabolic disorder due to biallelic pathogenic variants in the TH gene (*TH*) on 11p15.5, leading to decreased activity of the enzyme TH. This enzyme, a tetrahydrobiopterin (BH_4_)-dependent monooxygenase (BH_4_ is the essential cofactor for TH), catalyzes the rate-limiting step in catecholamine biosynthesis (Figure 1). Therefore, clinical symptoms and signs of TH deficiency are caused by dopamine deficiency and subsequent noradrenaline (norepinephrine) and adrenaline (epinephrine) deficits. The phenotypic spectrum of this rare metabolic disorder is exceedingly broad and differs according to age at onset, severity of disease, and responsiveness to levodopa (the precursor of dopamine).

**Figure 1:**
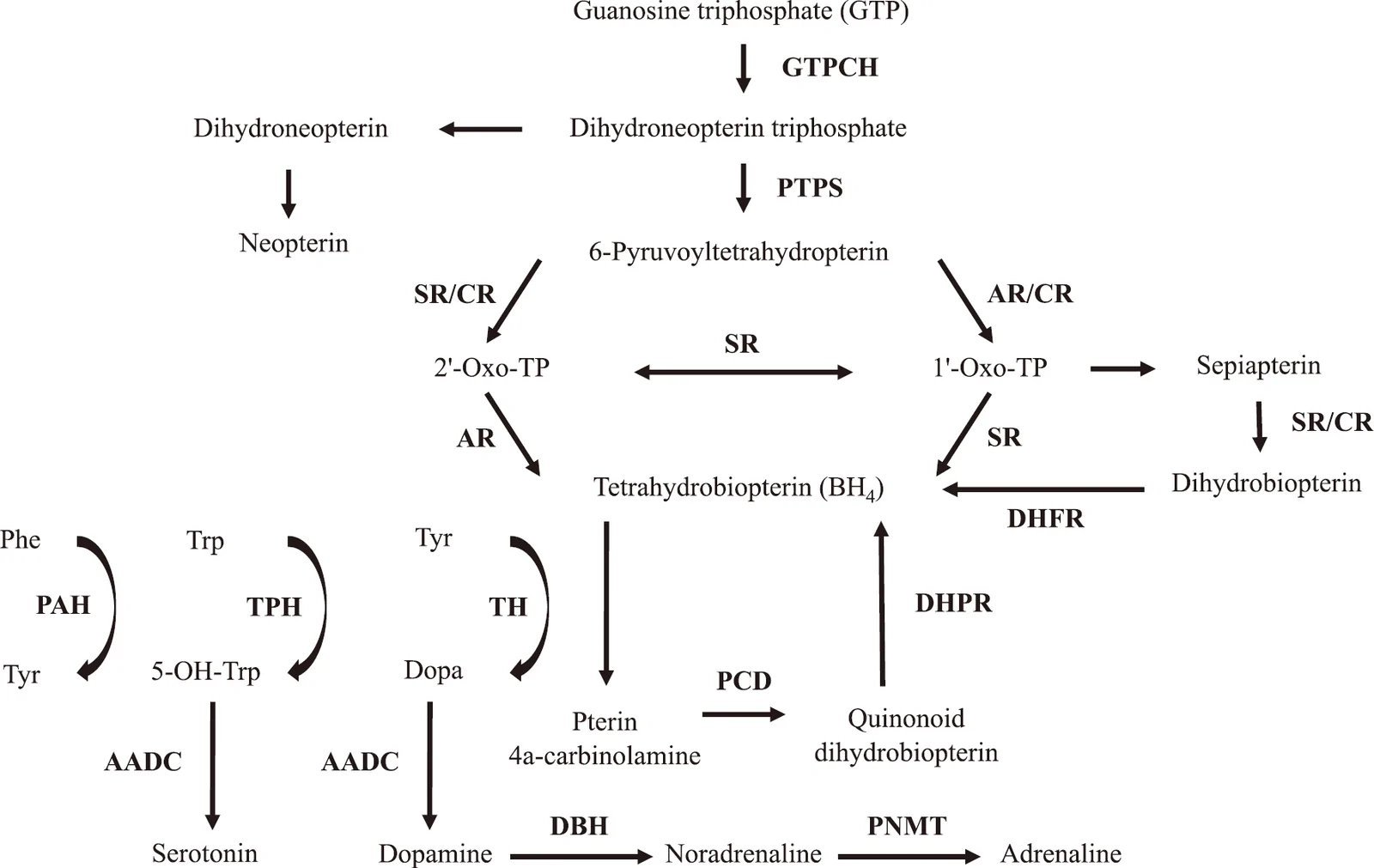
Biosynthesis of catecholamines and serotonin from aromatic amino acids and biosynthesis and regeneration of tetrahydrobiopterin (BH_4_) AADC = aromatic amino acid decarboxylase; AR = aldose reductase; CR = carbonyl reductase; DBH = dopamine beta-hydroxylase; DHFR = dihydrofolate reductase (DHFR activity in the salvage pathway is very low in the brain but is preserved in peripheral tissues); DHPR = dihydropteridine reductase; GTPCH = GTP cyclohydrolase 1; PAH = phenylalanine hydroxylase; PCD = pterin-4a-carbinolamine dehydratase; Phe = phenylalanine; PNMT = phenylethanolamine *N*-methyltransferase; PTPS = 6-pyruvoyltetrahydropterin synthase; SR = sepiapterin reductase; TH = tyrosine hydroxylase; TPH = tryptophan hydroxylase; Trp = tryptophan; Tyr = tyrosine; 1’-Oxo-TP = 2’-hydroxy-1’-oxopropyltetrahydrobiopterin; 2’-Oxo-TP = 1’-hydroxy-2’-oxopropyltetrahydrobiopterin; and 5-OH-Trp = 5-hydroxytriptophan.

The clinical spectrum is a continuum and generally the mild phenotype is TH-deficient dopa-responsive dystonia (DRD) and the severe phenotype is infantile parkinsonism/dystonia with motor delay or infantile encephalopathy with motor delay^1-17)^. Since clinical suspicion is a key to the diagnosis of TH deficiency, physicians should be aware of the wide phenotypic spectrum. Most patients with TH deficiency demonstrate positive response to oral administration of levodopa. However, the degree of responsiveness is extremely variable; a dramatic and sustained response (complete or near-complete responsiveness of symptoms) in mildly affected cases with DRD and a relatively good, moderate, or poor response in severely affected cases showing obvious motor development delay. When remarkable motor development delay is recognized in infants with TH deficiency, these patients are prone to levodopa-induced dyskinesias at the beginning of levodopa therapy and should be treated with very low initial doses of levodopa to avoid intolerable dyskinesias.

In this short review, I summarize these clinical and therapeutic features of TH deficiency and discuss possible underlying mechanisms of phenotypic heterogeneity.

## Clinical characteristics

Frequency of selected clinical features of TH deficiency is shown in Table 1.

**Table 1:** Clinical characteristics of tyrosine hydroxylase (TH) deficiency

Feature	% of patients with feature
Dystonia	60-85%
Motor development delay	50-75%
Truncal hypotonia	50-75%
Parkinsonian features	40-70%
Cognitive impairment	30-60%
Oculogyric crises	30-55%
Diurnal variation	30-40%
Pyramidal signs	30-40%
Autonomic symptoms	20-35%
Ptosis	15-20%
Seizures	< 5%

Data are from references 11 and 13-16 (ptosis is listed separately from autonomic symptoms in references 14 and 15).

### Movement disorders

Dystonia is the most common movement disorder in patients with TH deficiency and is mostly generalized and localized in lower limbs^13, 15)^. More than 45 cases with TH-deficient DRD (the mild phenotype of TH deficiency^7)^) have been reported. Onset of these DRD cases is generally between ages 12 months and 12 years; in addition, some adolescent- and adult- onset patients have been recognized^9, 12, 13, 15, 18-21)^. In general, initial symptoms of mildly affected patients are lower-limb dystonia and/or difficulty in walking. Typically, gradual progression to generalized dystonia occurs. In contrast to autosomal dominant GTP cyclohydrolase 1 (GTPCH [Figure 1]) deficiency (GTPCH-deficient DRD^12, 22)^ [DYT5a]), there is no predominance of clinically affected females in autosomal recessive TH deficiency (including TH-deficient DRD [DYT5b])^15, 23)^. Without levodopa treatment, most of the patients with TH deficiency, including those with TH-deficient DRD, become wheelchair bound; some had a misdiagnosis of cerebral palsy^5, 13, 15, 16, 19, 21, 23-26)^. Parkinsonian features (hypokinesia or bradykinesia, postural and/or resting tremor, rigidity, hypomimic face, etc.) are found frequently and myoclonus (including atypical myoclonus-dystonia^27)^) as well as other involuntary movements can be recognized in TH deficiency.

### Developmental delay and intellectual disability

Motor milestones in infancy (head control, rolling over, sitting without assistance, crawling, walking alone, etc.) are obviously delayed in severely affected cases with TH deficiency. More than 165 patients with the severe phenotype of TH deficiency have been reported; onset of these patients is generally before age 12 months^9, 12, 13, 15, 16, 19, 28)^.

Standardized neuropsychological examinations have not been conducted in most of the cases with TH deficiency and it is difficult to provide specific information on impaired cognitive status (i.e., global developmental delay in children younger than five years and intellectual disability in individuals aged five years and older^14)^) in this disorder. Patients who started levodopa treatment after two years of age tended to demonstrate cognitive dysfunction^29)^. Recently, a relatively low incidence of cognitive impairment (14%) has been found in a single-center retrospective analysis^16)^.

### Other neurologic features

Hypotonia is one of the most common initial symptoms in infantile- and neonatal-onset TH deficiency. Generally, tone is markedly decreased in the truncal muscles (axial hypotonia) and that in the extremities is variable; truncal hypotonia with limb hypertonia due to rigidity and/or spasticity is often recognized.

Oculogyric crises are dystonic episodes characterized by sustained, conjugate, and typically upward deviation of the eyes of variable duration. These episodes should be distinguished from tonic eye deviations observed in epileptic seizures.

Typical diurnal fluctuation of symptoms (worsening of the symptoms toward the evening and their alleviation in the morning after sleep^30)^) has been recognized in approximately 40% of patients with TH-deficient DRD (a much lower incidence than that in GTPCH-deficient DRD^12, 23)^). Although diurnal variation can also be observed in some severely affected patients, it tends to be less prominent and often atypical (e.g., diurnal variation of axial hypotonia but not of limb dystonia^31)^).

Many patients with TH deficiency have clinical features suggestive of pyramidal signs (hyperreflexia, spasticity, extensor plantar responses, etc.); plantar responses can become flexor after beginning levodopa therapy, suggesting that the previous findings may be consistent with a dystonic phenomenon (the striatal toe) rather than a Babinski response^23, 24, 32-34)^. Some cases were initially misdiagnosed with spastic paraparesis^15, 21)^.

Autonomic dysfunction can manifest with a variety of symptoms (palpebral ptosis, increased drooling, excessive sweating, nasal congestion, temperature instability, etc.); in some papers, ptosis is listed separately from autonomic symptoms^14, 15)^ (Table 1). Paroxysmal periods of lethargy (with prominent autonomic disturbances) alternated with irritability (lethargy-irritability crises) can be found in some severely affected cases with TH deficiency; in general, however, abnormalities in the maintenance of blood pressure have not been recognized^6, 8, 9, 14, 19, 35)^.

Seizures are uncommon in patients with TH deficiency. On occasion, oculogyric crises may be confused with seizures (see above); therefore, some had a misdiagnosis of epilepsy^15, 16, 19, 36, 37)^.

### Neurobehavioral/psychiatric manifestations

Behavioral and psychiatric symptoms (irritability, attention-deficit traits, autism, anxiety, depression, etc.) can be found in some cases with TH deficiency. One affected child was assessed with an Autism Diagnostic Observation Schedule-Module 2 and diagnosed with autism spectrum disorder^38)^; unfortunately, standardized neurobehavioral/psychiatric studies have not been conducted in most patients with TH deficiency.

### Anthropometric disturbances

Pre-/post-natal growth retardation affecting head circumference (microcephaly), height, and/or weight can be observed in some severely affected cases with TH deficiency; feeding difficulties in neonatal and infantile periods lead to growth failure^13, 14, 16, 39)^.

### Noteworthy episodic symptoms

At least eight patients with TH deficiency have demonstrated remarkable episodic symptoms^9, 34, 40-45)^. These symptoms are frequently induced and/or exacerbated by fever and/or infections. Episodic symptoms include dystonia, parkinsonism, truncal hypotonia, loss of developmental milestones, motor and language regression, and lethargy. Episodes are often triggered by fever (including fever after vaccination), viral infections, upper respiratory tract infections, and flu-like illness. Because there are various intervals between episodes, it is sometimes difficult to determine an exact effect of levodopa therapy to reduce these episodes; however, after starting levodopa treatment, no remarkable episodic recurrence occurs in most of the cases with this distinctive phenotype of TH deficiency. Even in patients with other phenotypes of TH deficiency, deterioration of symptoms by fever and/or infections can be observed. Therefore, fever and/or infections should be promptly treated to prevent possible worsening of symptoms in TH deficiency.

## Laboratory findings

The enzyme TH is mainly expressed in the brain as well as adrenal medulla, and thus direct measurement of its activity is not a diagnostic option. Precise determination of cerebrospinal fluid (CSF) concentrations of neurotransmitter metabolites (before starting levodopa treatment) is diagnostic of TH deficiency. Biochemical CSF profile typically demonstrates the following^3, 8, 9, 46-49)^: decreased homovanillic acid (HVA; a metabolite of dopamine), normal 5-hydroxyindoleacetic acid (5-HIAA; a metabolite of serotonin), reduced HVA/5-HIAA ratio, decreased 3-methoxy-4-hydroxy-phenylethyleneglycol (a metabolite of norepinephrine), normal total biopterin (BP; BP includes BH_4_, quinonoid dihydrobiopterin, dihydrobiopterin, and oxidized biopterin, and most of the brain BP exists as BH_4_^50, 51)^ [Figure 1]), and normal total neopterin (which consists of degradation products [dihydroneopterin and oxidized neopterin] of dihydroneopterin triphosphate^50, 51)^ [Figure 1]).

As dopamine is a prolactin-inhibiting factor, blood prolactin levels can be increased in dopamine-deficient disorders (including TH deficiency), but it is not a sensitive marker and its normal levels do not exclude TH deficiency^9, 35, 48, 52-55)^. On the other hand, blood prolactin measurement during and after puberty is recommended to screen for hyperprolactinemia as comorbidity^56)^.

## Neuroimaging

In most of the patients with TH deficiency, brain MRI is normal. Brain MRI demonstrated no abnormalities in the basal ganglia of two cases with TH-deficient DRD even 38 and 43 years after onset of the disorder^24)^. MRI revealed cortical atrophy, ventriculomegaly, and other non-specific brain changes in some patients. There were no correlations between these MRI changes and clinical phenotypes^11, 14, 15, 57)^.

## Neuropathology

In TH-deficient DRD^5, 24, 58)^ and typical GTPCH- deficient DRD^59-66)^, neuropathological investigations showed normal cell density and no Lewy bodies in the substantia nigra (SN). Characteristically, there were decreases of melanin-pigment from SN neurons without dopaminergic neuronal degeneration in autopsied cases with both disorders^67)^. There have been no reports of pathologic findings in severely affected patients with TH deficiency.

## Neurochemistry

There have been no reports of neurochemical data in TH-deficient DRD. However, similar to those in typical GTPCH-deficient DRD (TH protein levels were normal in the SN but markedly reduced in the striatum without degeneration of nigrostriatal dopaminergic neurons^59-62, 66)^), normal number of TH-positive neurons in the SN and severely decreased TH immunostaining, associated with normal dopamine transporter staining, in the striatum were found in a mouse model of mild TH deficiency (DRD *Th* knock-in mice)^68, 69)^.

Protein concentrations of dopaminergic markers, including TH, in the mesencephalon and pons were reduced in a 16-week-old miscarried human fetus having compound heterozygous *TH* variants^70)^; an elder sister of this fetus had the same pathogenic *TH* variants and developed the severe phenotype of TH deficiency (infantile encephalopathy with motor delay)^71)^. In a *Th* knock-in mouse model of very severe TH deficiency (encephalopathy *Th* knock-in mice being unresponsive to levodopa treatment), there was no loss of TH-immunoreactive neurons in the SN; recent quantitative analysis of fluorescence intensity in this model revealed moderate and severe TH protein decreases in the SN and striatum (40 % and 1% of wild type controls, respectively) without degeneration of nigrostriatal dopaminergic neurons^72, 73)^.

## Molecular diagnosis

The diagnosis of autosomal recessive TH deficiency can be established in a proband with suggestive clinical and/or laboratory findings by identification of biallelic pathogenic variants in *TH* by genetic testing. In Human Gene Mutation Database (HGMD Professional 2026.2), 113 pathogenic variants in *TH* (including 86 missense/nonsense mutations, 9 splicing substitutions, 3 regulatory substitutions, 5 small deletions, 1 small insertion, 2 small indels, 6 gross deletions, and 1 complex rearrangement) have been reported.

More than 95% of *TH* variants can be detected by sequence analyses; using whole-genome sequencing, one deep intronic variant^74)^ has been identified recently. Generally, to detect gross deletions and gross insertions/duplications, gene-targeted deletion/duplication analyses (including multiple ligation-dependent probe amplification, long-range PCR, quantitative PCR, etc.) should be conducted. Clinical features of four compound heterozygotes having pathogenic gross deletions have been reported^75-78)^. In addition, one heterozygote for a deletion of entire *TH*, who developed adult-onset Parkinson’s disease^79)^, has been included in the Human Gene Mutation Database.

Definite genotype-phenotype correlations have not been found in patients with TH deficiency. However, it has been reported that cases who were homozygous or compound heterozygous for a single- nucleotide variant in the promotor region of *TH* did not develop very severe TH deficiency^27, 75, 80-83)^. Once biallelic pathogenic variants in *TH* are identified in an affected family member, prenatal testing for a pregnancy at increased risk and preimplantation genetic testing are possible, and prenatal diagnoses of TH deficiency have been reported^71, 83, 84)^.

At least six individuals with monoallelic *TH* variants have been reported to develop muscle stiffness after exercise or dystonic symptoms responding to levodopa^15, 19, 33, 41, 85)^, suggesting that, as in the case of sepiapterin reductase (SR [Figure 1]) deficiency^86-88)^, heterozygous pathogenic variants in *TH* might cause autosomal dominant DRD. However, in these heterozygotes, there is still a possibility of undetermined structural or deep intronic *TH* variants on the other allele or of unknown additional variants of other genes relating to striatal dopamine content^15, 89)^.

### Differential diagnosis

The major differential diagnosis of TH deficiency is summarized in Table 2^12, 13, 47, 88, 90, 91)^. In general, patients with autosomal recessive BH_4_-related enzyme deficiencies, including recessively inherited severe GTPCH deficiency, develop hyperphenylalaninemia; an exception is autosomal recessive SR deficiency (in which BH_4_ can be synthesized through the salvage pathway in peripheral tissues [Figure 1]). Of note, some patients with TH deficiency are initially misdiagnosed as having cerebral palsy, spastic paraplegia, or epilepsy (see above).

**Table 2:** Differential diagnosis of tyrosine hydroxylase (TH) deficiency

	Features of disorder	
Disorder(s)/MOI	Overlapping with TH deficiency	Distinguishing from TH deficiency
GTPCH-deficient DRD/AD	Childhood-onset dystonia, parkinsonism, diurnal fluctuation of symptoms, etc. Dramatic & sustained response to levodopa.	Symptoms in TH deficiency, except for the mild phenotype (DRD), manifest earlier & are more severe than those in GTPCH-deficient DRD. Affected individuals show↓BP & NP in CSF^1^.
SR deficiency/AR	Associated with very wide phenotypic spectrum; from mild (DRD) to severe (motor & speech delay, truncal hypotonia, cognitive impairment, parkinsonism, oculogyric crises, etc.). Significant response to levodopa.	Sleep problems & psychiatric & behavioral abnormalities tend to be more remarkable in SR deficiency^2, 3^. SR deficiency causes loss of brain BH_4_ (without HPA) & has impaired serotonin biosynthesis. Affected individuals show↑sepiapterin in CSF^2^.
BH_4_ deficiencies associated with HPA/AR^4^	Motor & speech delay, truncal hypotonia, cognitive impairment, dystonia, parkinsonism, oculogyric crises, etc. Substantial response to levodopa.	These disorders have impaired phenylalanine homeostasis & serotonin biosynthesis. In general, these conditions present with HPA detected on newborn screening^2^.
AADC deficiency/AR	Motor & speech delay, truncal hypotonia, cognitive impairment, oculogyric crises, dystonia, behavioral problems, etc.	Except for specific variants in the levodopa binding site, patients with AADC deficiency do not respond to levodopa (without DCI).

AADC = Aromatic L-amino acid decarboxylase, AD = autosomal dominant, AR = autosomal recessive, BH_4_ = tetrahydrobiopterin, BP = total biopterin, CSF = cerebrospinal fluid, DCI = decarboxylase inhibitor, DRD = dopa-responsive dystonia, GTPCH = GTP cyclohydrolase 1, HPA = hyperphenylalaninemia, MOI = mode of inheritance, NP = total neopterin, SR = sepiapterin reductase, TH = tyrosine hydroxylase. 1. Furukawa and Kish. [1999]^47)^, 2. Opladen et al. [2020]^88)^, 3. Weissbach et al. [2022]^13)^, 4. AR BH_4_-deficient disorders with HPA include recessively inherited GTPCH, PTPS, PCD, and DHPR deficiencies [see Figure 1].

## Treatment

Treatment for TH deficiency is aimed at correcting dopamine deficiency (and subsequently noradrenaline and adrenaline deficits) (Table 3)^6, 8-10, 12-14, 16, 21, 29, 35, 44, 55, 92-95)^. Importantly, none of the symptoms and signs of TH deficiency improve without proper treatment with levodopa. Recently, Bondarenko and colleagues^14)^ described levodopa dose recommendations according to their classification of clinical phenotypes (DRD and infantile dystonia-parkinsonism) and stated that “Evidence for or against the use of medication other than levodopa is much scarcer in the literature, and thus recommendations are influenced by expert opinion.” I agree with them, but considering possible underlying mechanisms of phenotypic heterogeneity, in Table 3, I demonstrate levodopa dose recommendations for the mild phenotype (DRD) and the severe phenotype with motor delay (infantile parkinsonism/dystonia with motor delay and infantile encephalopathy with motor delay).

**Table 3:** Treatment for tyrosine hydroxylase (TH) deficiency

Type	Treatment	Dose/Consideration
Correction of dopamine deficiency	Levodopa (precursor of dopamine)	Suggested dose^1^^-^^10^ 1) Initial dose 2) Chronic dose Mild phenotype (DRD) 1) 1-2 mg/kg/day levodopa with a DCI (divided into 2-3 doses) 2) 2-10 mg/kg/day levodopa with a DCI Severe phenotype with motor delay 1) 0.1-1 mg/kg/day levodopa with a DCI (divided into 3-6 doses) 2) 0.5-35 mg/kg/day levodopa with a DCI Gradual titration (changing the dose slowly & by small increments according to tolerance; e.g., 0.1-0.5 mg/kg/day every month^4^) is recommended. When levodopa-induced dyskinesias occur, temporary dose reduction with later gradual increment & addition of selegiline^11^ or amantadine^12^ are recommended.
	Selegiline^11^ (MAO-B inhibitor: this medicine prevents the breakdown of dopamine)	Suggested dose^1^^-^^3, 8, 9, 13^^-^^19^ 1) Initial dose 2) Chronic dose Severe phenotype with motor delay 1) 0.1-0.2 mg/kg/day (or 0.5-5 mg/day) 2) 0.1-0.3 mg/kg/day (or 2.5-5 mg/day) Selegiline can be added to levodopa therapy in severely affected individuals not tolerating higher doses of levodopa. Insomnia is an important side effect of selegiline & this medicine should not be administered in the evening. Selegiline should be used carefully in children due to safety concerns; read the instructions before prescribing.

DRD = dopa-responsive dystonia, DCI = decarboxylase inhibitor, MAO = monoamine oxidase. 1. Willemsen et al. [2010]^8)^, 2. Yeung et al. [2011]^9)^, 3. Chi et al. [2012]^92)^, 4. Pons et al. [2013]^93)^, 5. Furukawa et al. [2022]^12)^, 6. Weissbach et al. [2022]^13)^, 7. Yao et al. [2022]^21)^, 8. Bondarenko et al. [2025]^14)^, 9. de Gusmao et al. [2025]^29)^, 10. Ban et al. [2026]^16)^, 11. Selective monoamine oxidase (MAO)-B inhibitor, 12. *N*-methyl-D-aspartate (NMDA) receptor antagonist, 13. Dionisi-Vici et al. [2000]^35)^, 14. Häussler et al. [2001]^94)^, 15. Hoffmann et al. [2003]^6)^, 16. Yosunkaya et al. [2010]^95)^, 17. Giovanniello et al. [2012]^10)^, 18. Leuzzi et al. [2017]^55)^, 19. Champagne et al. [2022]^44)^.

### Levodopa

Levodopa is the first-line drug for TH deficiency and all of the patients with TH deficiency should be treated appropriately with levodopa in combination with a peripheral decarboxylase inhibitor (carbidopa or benserazide) (Table 3).

Patients with TH-deficient DRD demonstrate a dramatic and sustained response to levodopa and an excellent response in the absence of any motor adverse effects (wearing-off and on-off phenomena and levodopa-induced dyskinesias) of chronic levodopa therapy for more than 30 years has been reported^5, 24, 58)^. In contrast, in severely affected patients, the responsiveness is frequently not complete and/or it takes several months or even years for the full effects of treatment to become established; at least 15 severely affected cases have shown poor or no response to levodopa^14-16)^.

Severely affected patients, who demonstrate remarkable motor development delay, are often hypersensitive to levodopa therapy and are prone to intolerable side effects (i.e., severe levodopa-induced dyskinesias which develop at initiation of levodopa treatment). Because of this hypersensitivity, these cases should be treated with extremely low initial doses of levodopa; gradual titration (changing the dose slowly and by small increments) to higher doses is strongly recommended.

### Other agents

A selective monoamine oxidase (MAO)-B inhibitor (selegiline), which prevents the breakdown of dopamine, has been added to levodopa therapy (or very rarely used as monotherapy) in more than 25 severely affected patients generally not tolerating higher doses of levodopa^6, 8-10, 16, 20, 27, 35, 41, 44, 55, 92, 94-98)^. Most of these cases have shown some improvement and selegiline can be considered as a supplementary treatment in combination with levodopa (Table 3).

As reported in compound heterozygotes for *GCH1* variants (who showed developmental motor delay, dystonia, parkinsonism, and hypersensitivity to levodopa treatment)^99)^, amantadine (which acts as a *N*-methyl-D-aspartate [NMDA] receptor antagonist and has dopaminergic and anticholinergic properties) has alleviated levodopa-induced dyskinesias in severely affected patients with TH deficiency^76, 93)^. Thus, amantadine can be considered as a supplementary treatment in combination with levodopa to suppress dose-related dyskinesias in patients with the severe phenotype.

Dopamine agonists, which directly stimulate postsynaptic dopamine receptors, have been reported to demonstrate limited or no beneficial effects in patients with TH deficiency^8, 27, 35, 55)^. On a theoretic basis, catechol-O-methyl transferase (COMT: this enzyme breaks down levodopa) inhibitors can be considered as a supplementary treatment in combination with levodopa, but there has been only one report describing a single case treated with COMT inhibitors^55)^. During chronic levodopa therapy, there is a possibility of the development of cerebral folate deficiency; in this case, folinic acid supplementation should be considered^76)^.

### Therapies under investigation

At the present time, no clinical trials for TH deficiency can be found in ClinicalTrials.gov in the US and EU Clinical Trials Register in Europe. Nygaard and colleagues have reported future treatment opportunities for TH deficiency^100)^.

### Pregnancy management

As indicated in GTPCH-deficient DRD^101)^, levodopa treatment should be continued in patients with TH deficiency during pregnancy^20, 102)^. Intensive supervision by a multidisciplinary team before, during, and after pregnancy is essential. Generally, it is better not to use selegiline during pregnancy due to concerns about potential vasoconstrictive effects.

### Agents/circumstances to avoid

In patients with TH deficiency, antipsychotic medications and other dopamine receptor antagonists (e.g., metoclopramide) are not recommended on a theoretic basis. Of great importance, abrupt discontinuation of levodopa and other dopaminergic medications should be avoided to prevent the potential development of dystonic crisis/storm or akinetic crisis.

## Pathogenesis

Because null variants of *TH* are lethal as shown in *TH* (-/-) knock-out mice^103)^, patients with recessively inherited TH-deficiency (both homozygotes and compound heterozygotes for *TH* pathogenic variants) appear to have some residual TH activity and the degree of this activity is related to the disease severity^104)^. For instance, a mutated recombinant enzyme in TH-deficient DRD showed approximately 15% and another one in TH-deficient infantile parkinsonism/dystonia with motor delay demon- strated 0.3-16% of wild-type enzyme activity^1-3)^. In addition, being similar to compound heterozygotes for *GCH1* variants (versus heterozygotes [DRD: DYT5a])^99, 105)^, severely affected patients with TH deficiency (versus mildly affected patients [DRD: DYT5b]) generally demonstrate obvious motor development delay, truncal hypotonia, and levodopa- induced dyskinesias at the beginning of levodopa therapy.

Nigrostriatal dopaminergic terminals seem to be the most vulnerable to the TH-deficient state (which may disrupt TH protein axonal transport from the soma) and the BH_4_-deficient state (which may impair the stability of TH molecules in the striatum)^61, 62, 73, 106-108)^. Recently, Roman and colleagues^109)^ found dysregulation of extracellular signal-related kinase phosphorylation in their DRD *Th* knock-in mice and have suggested that this dysregulated signaling may be caused by loss of D1 dopamine receptor stimulation resulting from early- life dopamine deficiency. In fact, D1-spiny projection neurons fail to undergo maturation of excitability in mice deficient for postnatal increases in striatal dopamine neurotransmission; in these mice, D2-spiny projection neurons mature normally^110)^. It is known that, in neurologically normal human subjects, striatal dopamine concentrations significantly increase during postnatal development (Figure 2) and decrease during aging^111)^. Moreover, levels of TH protein (Figure 2) and BP (most of the brain BP exists as BH_4_ [the natural cofactor for TH]) in the normal human striatum also significantly increase postnatally^111, 112)^. Thus, there is a possibility that mildly affected patients with DRD due to recessively inherited TH deficiency and dominantly inherited GTPCH deficiency both manifest their characteristic dystonia through dysfunctional D1 dopamine receptors caused by congenital dopamine loss and a lack of sufficient postnatal dopamine increases in the striatum.

**Figure 2:**
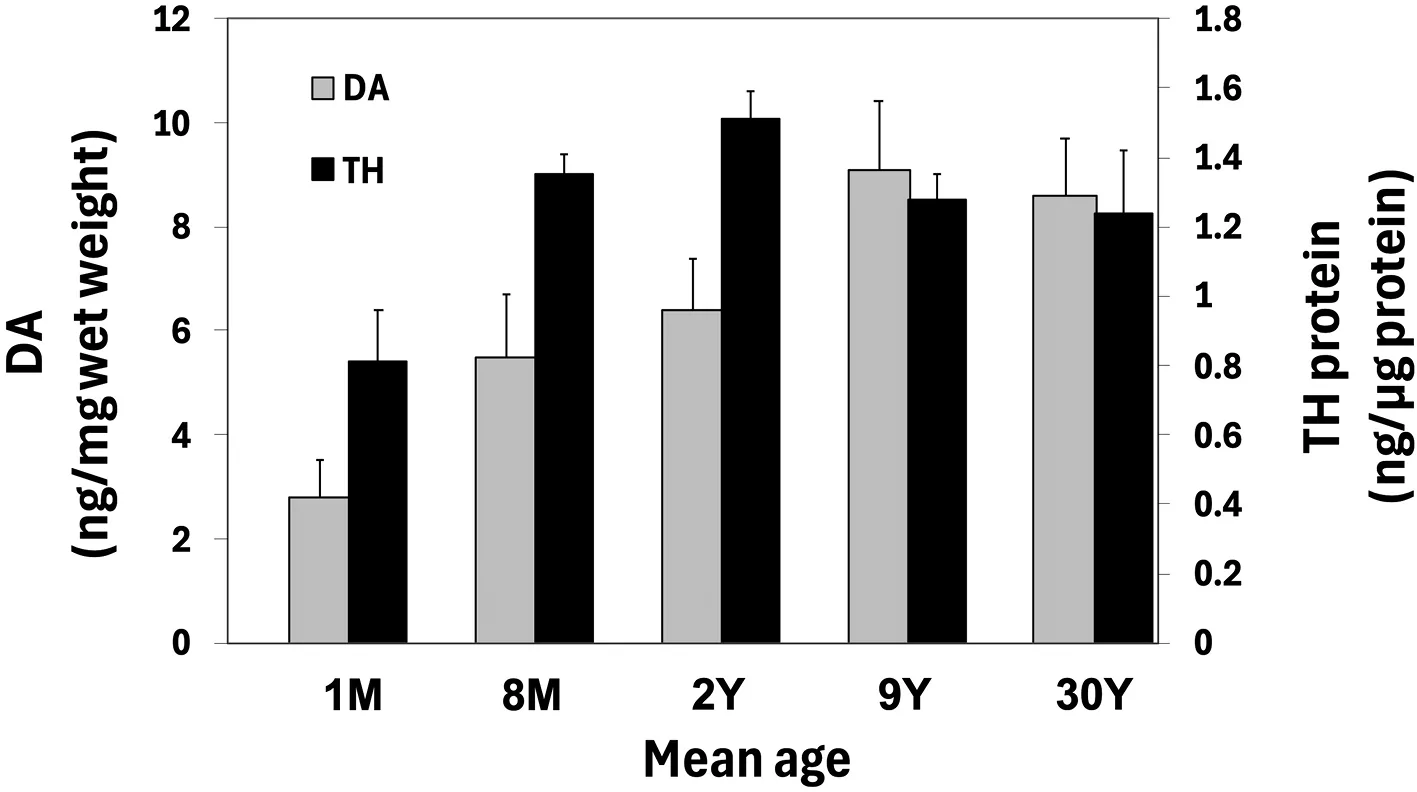
Influence of development on dopamine (DA) and tyrosine hydroxylase (TH) protein levels in autopsied human putamen Values are expressed as means ± SEM. In the putamen of 35 neurologically and neuropathologically normal subjects, TH protein and DA concentrations significantly increased during postnatal development and reached maximal levels in the 2-year-group and in the 9-year group, respectively; the 35 normal subjects were separated into the 5 groups (n = 7 each) with mean ages of 1 month (mean ± SEM, 0.08 ± 0.04 years; range, 1 day-3.5 months), 8 months (0.65 ± 0.07; 4-10 months), 2 years (1.8 ± 0.2; 1.0-2.8 years), 9 years (8.5 ± 1.6; 3.5-14), and 30 years (30 ± 3; 17-41) (Data are from reference 111).

In the autopsied patient with TH-deficient DRD, who demonstrated a dramatic and sustained response to low doses of levodopa for more than 30 years, normal cell density in the SN has been reported^5, 24, 58)^. Although there was no loss of neuronal volume, decreased dopaminergic markers, including TH, in the mesencephalon were found in the very severely affected patient with TH deficiency^70, 71)^. In this patient, cortical neurodevelopmental markers were also reduced, indicating abnormalities of synaptic and brain maturation. Similarly, in the encephalopathy *Th* knock-in mice (which were unresponsive to levodopa treatment), quantitative analysis of fluorescence intensity showed TH protein loss not only in the SN (40% of wild type controls) and striatum (1%) but also in other brain areas including the cortex (22%), whereas there was no dopaminergic neuronal degeneration^72, 73)^. In these very severely affected mice, interactions between TH neurons and inhibitory neuronal populations in the motor cortex appeared to be disruptive, potentially contributing to levodopa-unresponsive impairments.

## Conclusion

Since the discovery of various *TH* pathogenic variants responsible for TH deficiency, our understanding of this recessively inherited metabolic disorder has exceedingly increased. However, the exact underlying mechanisms of phenotypic heterogeneity in patients with TH deficiency are still unknown. The normal human brain data indicate that development of the nigrostriatal dopaminergic system (including BH_4_, TH, and dopamine biosyntheses) is incomplete at birth and striatal dopamine levels significantly increase during postnatal development. Because of congenital dopamine deficiency and insufficient postnatal dopamine increases in the striatum, mildly affected patients with TH deficiency may develop DRD through dysfunctional D1 dopamine receptors. In addition, more severely affected patients may demonstrate remarkable motor development delay in infancy and hypersensitivity to levodopa therapy caused by profound brain dopamine deficit (without dopaminergic neuronal degeneration) and consequent abnormal synaptic and brain maturation. Considering such an important role of dopamine in postnatal development of the brain, deficiency of this neurotransmitter should be corrected by proper treatment with levodopa (the precursor of dopamine) as soon as genetically or biochemically diagnosed or upon the appearance of characteristic symptoms and signs of TH deficiency. Thus, physicians should know the broad phenotypic spectrum of this treatable metabolic disorder and a therapeutic trial of levodopa is still an important practical approach to the diagnosis.

## Author contributions

YF wrote the manuscript and approved the final version.

## Conflicts of interest statement

The author declares that there are no conflicts of interest.
